# Wound Healing and Anti-Inflammatory Effect in Animal Models of *Calendula officinalis* L. Growing in Brazil

**DOI:** 10.1155/2012/375671

**Published:** 2012-01-24

**Authors:** Leila Maria Leal Parente, Ruy de Souza Lino Júnior, Leonice Manrique Faustino Tresvenzol, Marina Clare Vinaud, José Realino de Paula, Neusa Margarida Paulo

**Affiliations:** ^1^Laboratory of Natural Products, Pharmacy Faculty, Federal University of Goiás, 74605-220 Goiânia, GO, Brazil; ^2^Department of General Pathology, Institute of Tropical Pathology and Public Health, Federal University of Goiás, 74605-050 Goiânia, GO, Brazil; ^3^Veterinary School, Federal University of Goiás, 74001-970 Goiânia, GO, Brazil

## Abstract

*Calendula officinalis* is an annual herb from Mediterranean origin which is popularly used in wound healing and as an anti-inflammatory agent. In this study, the ethanolic extract, the dichloromethane, and hexanic fractions of the flowers from plants growing in Brazil were produced. The angiogenic activity of the extract and fractions was evaluated through the chorioallantoic membrane and cutaneous wounds in rat models. The healing activity of the extract was evaluated by the same cutaneous wounds model through macroscopic, morphometric, histopathologic, and immunohistochemical analysis. The antibacterial activity of the extract and fractions was also evaluated. This experimental study revealed that *C. officinalis* presented anti-inflammatory and antibacterial activities as well as angiogenic and fibroplastic properties acting in a positive way on the inflammatory and proliferative phases of the healing process.

## 1. Introduction

*Calendula officinalis *L. (Compositae) is an annual herb from Mediterranean origin. It is believed that it has been introduced in England during the 13th century [1] and found in Europe as a cultured plant. Wild plants are rare [2]. Its medicinal use seemed to be most diffused from the 13th century and especially in the wound healing aspect. It was used as balm and creams, as antiseptic and anti-inflammatory agents during the north-American civil war as well as in the first World War [2, 3].

The flowers of *C. officinalis* are found in chapters which may distinguish the varieties through its color and size. They have medicinal use especially as an anti-inflammatory agent, for the treatment of wound, first-degree burns, contusions, and skin rashes. The German sanitary authorities recommended its topic use in leg ulcers and its internal use only against inflammatory lesions in the oral and pharynx mucosae [4–6]. The main chemical components found in the flowers are saponins, triterpenes, alcohol triterpenes, fatty acid esters, carotenoids, flavonoids, coumarines, essential oils, hydrocarbons, and fatty acids [7–9].

The anti-inflammatory activity of *C. officinalis *flowers cultured in Europe and Asia has been evaluated and evidenced through the model of edema induction of the ear through croton oil and the model of edema induction of the paw through carrageenan [2, 3, 7, 10–12]. The angiogenic activity of the aqueous extract of *C. officinalis *cultured in England was also evidenced [13].

Most of the literatures that report the medicinal activity of *C. officinalis* were performed in European or Asian countries from plants cultured in those locations. The different conditions of culturing a plant may alter some specific patterns of the vegetal metabolism which may activate or inactivate some metabolic pathways [14]. This study aimed at the evaluation of the wound healing and antibacterial activities of the ethanolic extract and its fractions from the flowers of *C. officinalis* cultured in Brazil (Parana estate).

## 2. Material and Methods

### 2.1. Plant Material

Dried and pulverized flowers of *C. officinalis *collected in Parana estate, Brazil, during the month of February, 2006, were commercially obtained from the company Empresa Clorofila (Goiania, GO, Brazil). To the quality control of the plant material, a pharmacognostic evaluation was performed which analyzed the organoleptic properties, ashes microscopy, and the ashes contents. Also the total flavonoids content dosages and thin-layer chromatography analysis were performed [3, 15].

To obtain the ethanolic extract (CEE), the pulverized flowers were extracted with 96° GL PA ethanol by cold maceration and concentrated by rotary evaporator at 40°C. To obtain of the hexanic and dichloromethane fractions, the CEE was soluble into a MeOH/H_2_O (7 : 1) mix followed by an extraction with hexane and afterwards with dichloromethane. The resulting extracts which were hexanic (HCF) and dichloromethane (DCF) were concentrated by rotary evaporator at 40°C.

As the ethanolic extract, the hexanic and dichloromethane fractions were insoluble in distilled water, solutions of 1% of the extract and fractions previously diluted in 70% ethanol were prepared and then used to test the angiogenic activity of the CAM. To test the angiogenic activity of the ethanolic extract on cutaneous wounds in rats, an aqueous solution of 1% of the extract was daily prepared.

### 2.2. Experimental Animals

36 *Wistar* female rats, 60 days old, weighing between 160 and 190 g-interval were used. The animals were adapted during a ten-day period and maintained in individual polyethylene cages covered with coarse saw dust under controlled environmental conditions such as room temperature of 23 ± 2°C, relative air humidity between 50 and 70%, and photoperiod light/dark of 12 h. Water and ration were supplied *ad libitum.*

75 fertilized eggs from Cobb chickens, two days old, were used and obtained from matrixes with age varying from 34 to 35 weeks old. We followed the ethical principles in animal experimentation recommended by the National Council for Control of Animal for Experiments (CONCEA).

The experimental protocol of the animals used in this study was evaluated and approved by the Ethics in Research Committee of the Federal University of Goias (protocol number 019/2007).

### 2.3. Chorioallantoic Membrane Model (CAM)

#### 2.3.1. Preparation of the CAM

The CAM was prepared aiming the angiogenic activity evaluation according to an adapted methodology [16]. The eggs were randomly distributed into four groups with 15 eggs each and submitted to the following treatments: SC1: solvent control 1 (70% ethanol); PC: positive control at 1% (17 *β*-estradiol); SC2: solvent control 2 (distilled water); CEE: ethanolic extract at 1%; DCF: dichloromethane fraction at 1%; HCF: hexanic fraction at 1%.

According to previously established protocols [16], the opening of the egg shell, the removal of the albumen, and the treatments were performed inside a type II security biological chamber. On the third day of incubation, through a small opening of the egg shell, 2 to 3 mL of albumen were removed followed by the sealing of the orifice with histological paraffin in fusion point. The incubation continued until the 10th day when the eggs were treated with 0.1 mL of the solutions described above which were instilled above the CAM surface. The eggs were sealed again and then incubated for two days.

After the euthanasia of the embryos, through medullar section of the atlantoocciptal region, fragments of the CAM from each egg were collected to morphometric analysis and quantification of the blood vessels.

#### 2.3.2. Morphometric Evaluation of the CAM

To the morphometric evaluation of the CAM, a fresh fragment was collected from 10 eggs from each group and distended on a glass slide and microscopically analyzed.

The microscopical analysis was performed with a digital image analysis system. The percentage of the vascular area per field was calculated through the Image J 1.3.1 (NIH, USA) software. 30 random fields were photographed and analyzed per sample, and this number was determined by accumulated mean [17].

#### 2.3.3. Evaluation of the Inflammatory Cells and the Number of Blood Vessels in the CAM

The membranes were fixed in tamponated formalin, processed and blocked into paraffin, and then sectioned into 5 micrometer sections and stained with hematoxylin and eosin (HE). 20 randomly chosen fields were photographed, number determined by accumulated mean. The inflammatory cells found in the CAM were classified in a qualitative form into absent (0/3), discrete (1/3), moderate (2/3), and accentuated (3/3).

The counting of the blood vessels in mesoderm from the CAM was performed through planimetry by counting of the points by using the GIM; P 2.4.3 software. A square reticulum composed by 25 points was overlapped on top of the histologic image [18, 19].

### 2.4. Cutaneous Wounds in Rats Model

The animals were weighted and randomly divided in two groups (*n* = 18) and subdivided in three subgroups (*n* = 6). The groups were evaluated in determined moments of the postoperatory (PO) period according to the protocol previously established by Lopes et al. [20] and Garros et al. [21] aiming of the evaluation of the initial phases of the inflammatory process (4 and 7 PO days) and the proliferative phase of the inflammatory process (14 PO days) resulting in a general evaluation of the healing. The groups are namely:

**Group 1**

Control (C), animals were treated with distilled water. C1 was evaluated in the 4th PO day, C2 in the 7th PO day, and C3 in the 14th PO day.

**Group 2**

CEE, animals were treated with aqueous solution of the ethanolic extract at 1%. CEE1 evaluated in the 4th PO day, CEE2 in the 7th PO day and CEE3 in the 14th PO day.

To the wound induction, a circular methalic punch of 1 cm of diameter was used on the dorsocervical region of each animal. The anesthetics consisted of the association between ketamin (70 mg/Kg, IM) and xylazine (10 mg/Kg, IM). Right after the surgery and daily afterwards on the same hour, 100 *μ*L of the CEE solution and of the solvent (distilled water) were instilled on the animals wounds.

Each animal was daily examined as to their general aspect, and the macroscopic evaluation of the wound was performed. The animals were euthanized in a CO_2_ chamber at 4, 7, and 14 PO days accordingly to previous protocols by Lopes et al. [20] and Garros et al. [21]. Fragments of the cutaneous wounds were collected to the histopathologic and immunohistochemical analysis.

#### 2.4.1. Histopathologic and Morphometric Evaluation

The morphometric analysis of the wounds was performed through the photographed images of the wounds at days zero, 4, 7, and 14 PO aiming of the determination of the contraction of the wound area [22].

The histopathologic analysis was performed after the fragments of the wounds were fixed with 10% buffered formalin, processed, and blocked with paraffin, and then sectioned into 5 micrometer sections and stained with hematoxylin and eosin (HE). The sections were also stained with Picrossirius to the collagen quantification under polarized light [23] through the calculus of the percentage of the marked area in green or reddish-yellow by field by using the IMAGE J 1.3.1 (NIH, USA) software.

The histopathologic analysis at 4, 7, and 14 PO days determined the healing phases of the wound through histologic variables such as the presence of fibrin, hemorrhage, hyperemia, inflammatory infiltration, reepithelialization, and epithelial hyperplasia [24].

#### 2.4.2. Angiogenic Activity Evaluation through Immunohistochemistry

Sections were incubated with bovine serum albumin (BSA), the blockage of endogenous peroxidase was performed, and the sections were incubated with the vascular endothelial growth factor (VEGF) primary antibody (147) (Santa Cruz Biotechnology-507), 1 : 500 dilution in a wet chamber, overnight at 4°C.

After this step, the streptavidin-biotin-peroxidase complex (kit LSAB-Dako K0690) was instilled over the sections for 20 minutes each reaction in a wet chamber at room temperature. The reaction was revealed with diaminobenzidine (DAB) solution, for 1 minute. A PBS solution was used for washing between the steps. The sections were counterstained with Mayer's hematoxylin, for 30 seconds, and the slides were mounted with synthetic resin (Sigma Aldrich, USA) and histological coverslips.

The counting of the blood vessels was performed through planimetry, and the intensity of the VEGF expression into the endothelial cells was evaluated.

### 2.5. *In Vitro* Antibacterial Activity

To determine the minimum inhibitory concentration (MIC), the cultures of *Staphylococcus aureus *(ATCC 6532), *Staphylococcus aureus *(ATCC 13048), *Micrococcus roseus *(ATCC 1740), *Micrococcus luteus *(ATCC 9341), *Bacillus cereus *(ATCC 14576), *Bacillus stearothermophylus (ATCC* 1262), *Enterobacter cloacae* (HMA/FTA 502), *Enterobacter aerogenes *(ATCC 13048), *Escherichia coli* (ATCC 25922), *Pseudomonas aeruginosa *(ATCC 9027), and *Serratia marcescens *(ATCC 14756) were used through the plaque assay method. The microorganisms were reactivated and processed according to NCCLS [25].

The MIC was determined through the plaque assay method when 1000 mg of the CEE, 500 mg of the HCF, and 350 mg of the DHC were incubated in essay tubes with 1.0 mL of DMSO.

### 2.6. Statistical Analysis

The results were submitted to the statistical analysis through the GraphPad InStat programme (Version 3.05 for Windows). From the Kolmogorov-Smirnov normality test, the morphometric data from the CAM were evaluated through the Kruskal-Wallis test followed by the Dunn posttest. The counting of the blood vessels was analyzed through ANOVA and the Tukey test. The histopatologic and immunohistochemical variables were analyzed through the Mann-Whitney test. The significance level was of *P* < 0.05.

## 3. Results and Discussion

The pulverized flowers of *C. officinalis* presented a yellowish coloring and soft and mildly aromatic odor. The microscopic analysis of the dust through the Steinmetz and Etzold staining evidence its structures: biseriate multicellular trichome of the corolla tube from the ligulate flower, ligule epidermal with striated cuticle and ligule parenchyma containing droplets of oil and pollen grains.

The total content of the ashes was of 8.47%. The thin layer chromatography evidenced the presence of rutin (Rf-0.47), flavonoids (Rf-0.29), and aglycone flavonoids (Rf-0.92). The contents of total flavonoids from the pulverized flowers were of 0.77% and in the CEE of 1.29%. WHO monographs [3] describe that *C. officinalis* should not present less than 0.4% of total flavonoids calculated as hyperoside up until 10% of the total ashes.

All data obtained from the pharmacognostic analysis are in accordance to what is described in the literature [3, 15] which indicates that the plant material tested corresponded to the pulverized flowers of *C. officinalis.*

The evaluation of the angiogenic activity in the CAM showed through the morphometric analysis an increase in the vascular area in the positive control, ethanolic extract, dichloromethane fraction, and hexane fraction groups when compared to the solvent control 1 (Table 1).

The quantification of the blood vessels performed through planimetry of the CAM stained with H&E and treated with positive control, ethanolic extract, dichloromethane fraction, and hexane fraction evidenced an increase in the number of blood vessels when compared to the solvent control 1 group (Table 2).

When substances are administered on the surface of the CAM, they may induce nonspecific inflammatory reactions leading to a secondary vessel proliferative response [26]. In this study, the CAM treated with *C. officinalis* in its extract or fraction forms presented a discrete inflammatory infiltration. We may infer that the increase in the neovascularization observed in the CAM is due to the direct acting of the *C. officinalis* and its fractions and is not related to the inflammatory reaction which is in accordance to similar findings by Patrick et al. [13]. Although there are reports in the literature [13] concerning the angiogenic activity of *C. officinalis, *there were not found descriptions of which of its component is responsible for this specific activity.

In the evaluation of the wound healing activity of *C. officinalis,* only the ethanolic extract was used because the extract and the fractions presented similar angiogenic properties.

The macroscopic evaluation of the wounds did not show purulent exudate in any of the wounded animals, but it was possible to observe a serous exudation in the animals from the CEE group up until the 4th PO day and in the control group up until the 7th PO day. The crusts started to form from the 3rd day, and, in the CEE group, they presented thinner and wetter when compared to the control group which were thicker and drier. At the 7th PO day, the crusts began to detach showing signs of epithelialization of the wounds, and, in the 14th PO day, the complete healing was verified on both groups.

**Figure 1:**
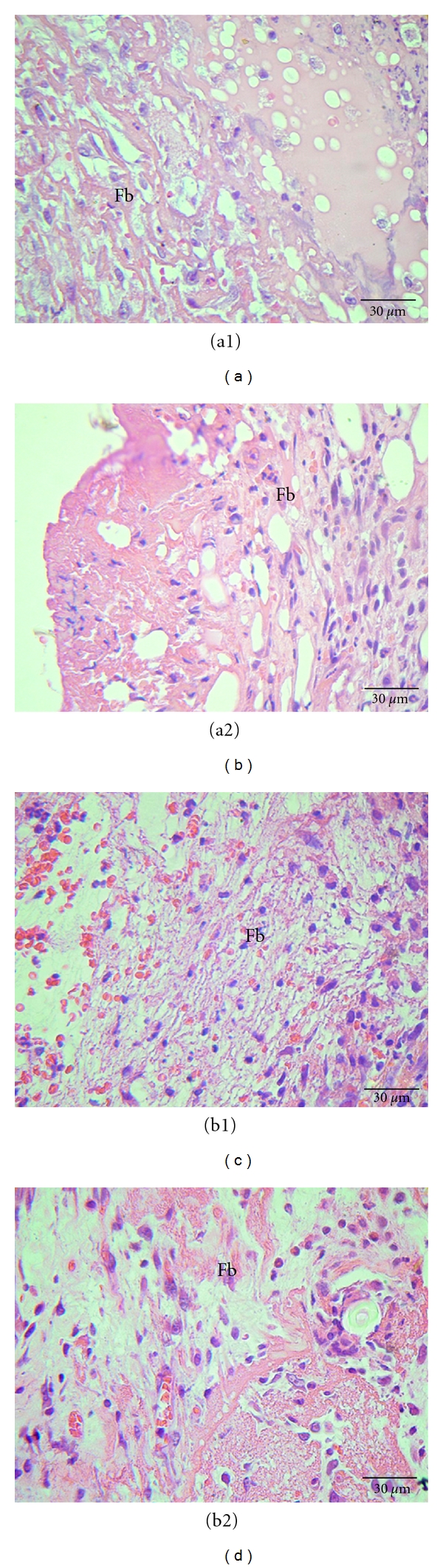
Photomicrograph of cutaneous wounds in rats at 4 (a1, a2) and 7 (b1, b2) PO days highlighting the presence of fibrin (Fb). (a1) and (b1) refer to the control group, treated with distilled water. (a2) and (b2) refer to CEE group. HE staining.

The microscopic evaluation of the CEE group at 4 and 7 PO days showed a significant decrease as to the presence of fibrin (median = 1.0 and 2.0, resp.)and hyperemia (median = 1.0 and 1.5, resp.) when compared to the control group at the same PO days (median = 3.0 and 2.5, resp.) (Figure 1). As to the evaluation of the other variables such as hemorrhage, inflammatory infiltration, reepithelialization, and epithelial hyperplasia, there was not a significant difference found between the analyzed groups.

Previous studies evaluated and evidenced the anti-inflammatory activity of *C. officinalis* and related this activity to the presence of triterpenes, especially to faradiol esters and taraxasterol [2, 3, 7, 10–12]. In this study, the same effect was evidenced due to the observation of the significant difference of the presence of fibrin and hyperemia found which represents the circulatory alterations directly related to the inflammatory phase of the healing process.

**Figure 2:**
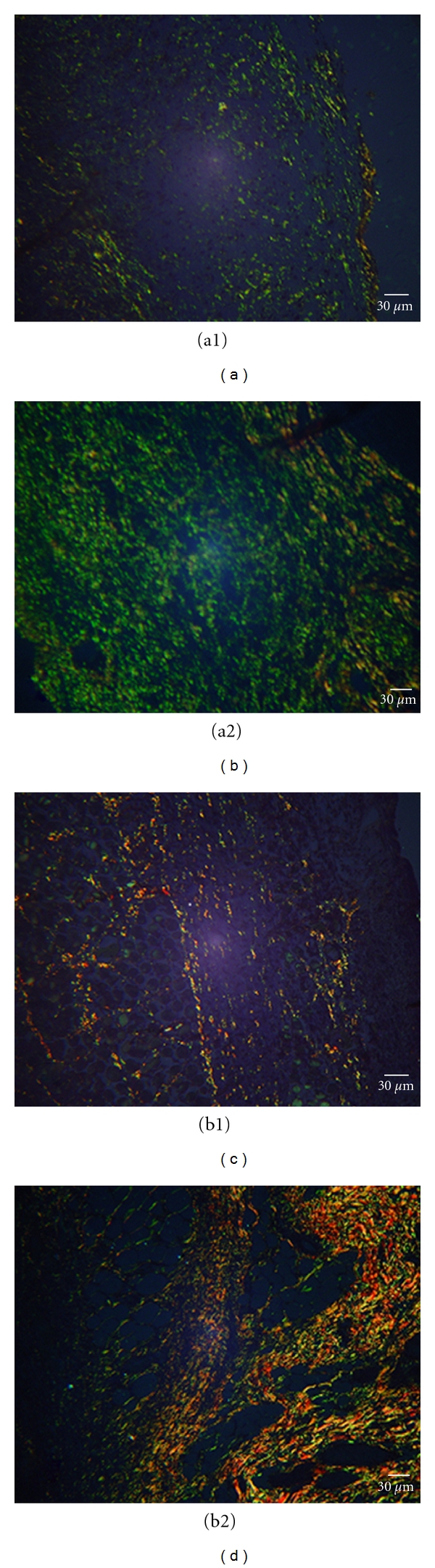
Photomicrograph of the cutaneous wound in rats (*n* = 6) at 4 (a1, a2) and 7 (b1, b2) PO days. (a1) Control group, treated with distilled water, type III collagen production evidenced through green polarized light; (a2) CEE group, type III collagen intense production; (b1) control group, type I collagen production evidenced by orange-yellow polarized light; (b2) CEE group, intense type I collagen production. Picrossirius staining.

It was possible to observe a significant increase in the collagen amount in the CEE group at 4 and 7 PO days (median = 8.27 and 6.33, resp.) when compared to the control group (median = 5.33 and 4.31, resp.) which indicates fibroplasia (Figure 2). On the 4th PO day, it was possible to observe the brilliant green coloration of the collagen on both groups, and, on the 7th PO day, the reddish-yellow one especially on the CEE group.

There are no reports found in the literature on the collagen mensuration through Picrossirius staining of cutaneous wounds treated with *C. officinalis.* In this study, the statistical confirmation of the increase in the collagen concentration in the wounds treated with the CEE at 4 and 7 PO days indicates that the extract contributed to the greater synthesis of this fiber. This indicates a positive role of the extract on the cutaneous wounds healing process. The triterpene faradiol palmitic ester stimulated the proliferation and migration of fibroblasts in previous *in vitro* studies [27]; we believe that this may have occurred in our study, stimulating the fibroplasias evidenced here.

**Figure 3:**
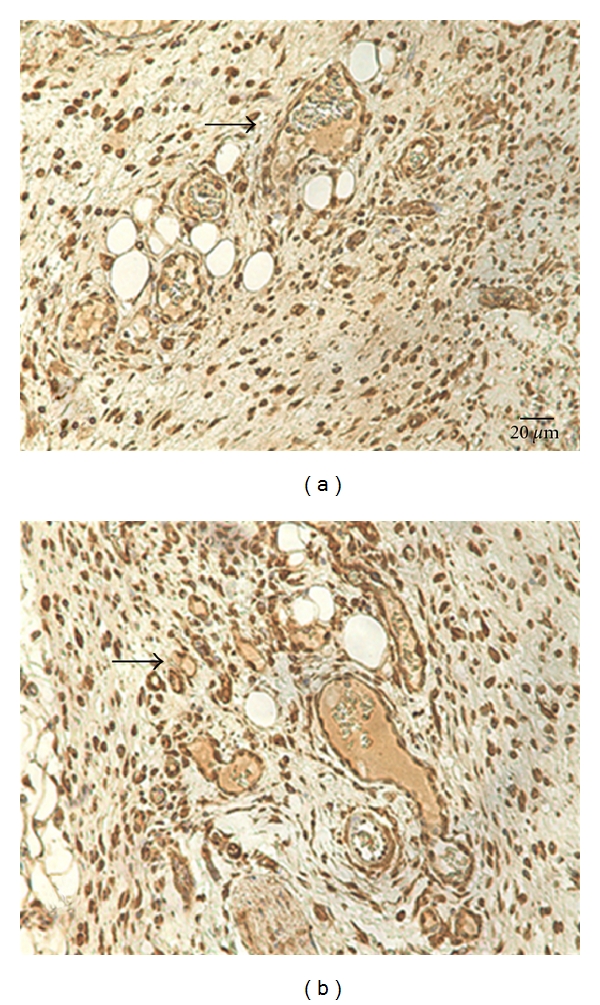
Photomicrograph from the dermis of rats at 7th PO days (*n* = 6). (a) Solvent control with distilled water. (b) EEC at 1%. Arrows indicate the VEGF positive marking on the dermis from both groups. Immunohistochemistry, 400x.

 The immunohistochemical evaluation showed an increase in the number of blood vessels in the dermis of the rats treated with CEE as it had a positive marking for VEGF (Figure 3). This finding confirms the intense activity of the extract on the neovascularization also observed on the CAM model.

The immunohistochemistry technique is a viable tool to angiogenesis evaluation through VEGF marking in this experimental model. Our data indicated that the CEE angiogenic property is not directly related to the increase of the intensity of expression of this marker. Other pro-aniogenic factors such as fibroblast growth factor (FGF), angiogenic cytokines as interleukin 8 (IL-8), tumor necrosis factor-alfa (TNF-*α*), and transformation growth factor-beta (TGF-*β*) [28, 29] may be related to the angiogenic activity evidenced in the *C. officinalis* extract by this study.

The antibacterial activity of the CEE and the hexanic fraction was observed against Gram-positive bacteria, and this effect was not observed on the dichloromethane fraction. The lowest concentrations of the extracts that inhibit microorganism's growth were CEE, MIC of 0.39 mg/mL against *S. aureus 13048* and *B. stearo thermophylus*; FDC, MIC of 4.37 mg/mL against *S. aureus 6532 *and* S. aureus 13048, *MIC of 1.08 mg/mL against *B. stearo thermophylus *and *B. cereus*, MIC of 0.5 mg/mL against *M. roseus;* FHC, MIC of 0.19 mg/mL against *S. aureus* 13048. According to Alonso [2], the antibacterial activity is due to the presence of flavonoids and essential oils in *C. officinalis.*

In the case of wound contaminations, there is the formation of a great quantity of exudate and toxin productions which may retard the healing process [30] in addition to the risk of presenting an entrance pathway to systemic infections. The antibacterial activity of *C. officinalis *evidenced in this study corroborates with the healing activity detected by this plant in this study as it prevents secondary infections of the wounds that can aggravate the injury.

## 4. Conclusions

*Calendula officinalis *growing in Brazil presented anti-inflammatory and antibacterial activities as well as the capability of stimulating fibroplasia and angiogenesis. Therefore, the *C. officinalis* extracts act in a positive form on the inflammatory and proliferative phases of the healing process of cutaneous wounds.

## Figures and Tables

**Table 1:** Morphometry (median, minimum/maximum) of the blood vessels from the fresh chorioallantoic membrane from fertilized chicken eggs.

Treatment	Marked area of the CAM
CEE	10.47 (1.43/57.49)*
HCF	9.33 (1.61/71.22)*
DCF	9.11 (2.26/54.35)*
PC	8.11 (3.56/20.64)*
SC1	5.10 (0.61/49.83)
SC2	5.78 (1.20/40.37)

CAM: chorioallantoic membrane, CEE: ethanolic extract of flowers from *Calendula officinalis *at 1%, HCF: hexanic fraction at 1%, DCF: dichloromethane fraction at 1%, PC: positive control at 1% (17 *β*-estradiol), SC1: solvent control with 70% ethanol, SC2: solvent control with distilled water. *P < 0.05 when compared to SC1.

**Table 2:** Influence of the *Calendula officinalis* extract and fractions on the number of blood vessels (median, minimum/maximum) of the chorioallantoic membrane from fertilized chicken eggs.

Treatment	Number of blood vessels
CEE	1 (0/4)*
HCF	1 (0/4)*
DCF	2 (0/13)*
PC	2 (0/13)*
SC1	1 (0/3)
SC2	1 (0/3)

CEE: ethanolic extract of flowers from *Calendula officinalis *at 1%, HCF: hexanic fraction at 1%, DCF: dichloromethane fraction at 1%, PC: positive control at 1% (17 *β*-estradiol), SC1: solvent control with 70% ethanol, SC2: solvent control with distilled water. *P < 0.05 when compared to SC1.
